# MicroRNA-122 Down-Regulation Is Involved in Phenobarbital-Mediated Activation of the Constitutive Androstane Receptor

**DOI:** 10.1371/journal.pone.0041291

**Published:** 2012-07-18

**Authors:** Ryota Shizu, Sawako Shindo, Takemi Yoshida, Satoshi Numazawa

**Affiliations:** Department of Biochemical Toxicology, Showa University School of Pharmacy, Tokyo, Japan; Wayne State University, United States of America

## Abstract

Constitutive androstane receptor (CAR) is a nuclear receptor that regulates the transcription of target genes, including CYP2B and 3A. Phenobarbital activates CAR, at least in part, in an AMP-activated protein kinase (AMPK)-dependent manner. However, the precise mechanisms underlying phenobarbital activation of AMPK are still unclear. In the present study, it was demonstrated that phenobarbital administration to mice decreases hepatic miR-122, a liver-enriched microRNA involved in both hepatic differentiation and function. The time-course change in the phenobarbital-mediated down-regulation of miR-122 was inversely correlated with AMPK activation. Phenobarbital decreased primary miR-122 to approximately 25% of the basal level as early as 1 h and suppressed transactivity of *mir-122* promoter in HuH-7 cells, suggesting that the down-regulation occurred at the transcriptional level. AMPK activation by metformin or 5-aminoimidazole-4-carboxamide 1-β-*D*-ribonucleoside had no evident effect on miR-122 levels. An inhibitory RNA specific for miR-122 increased activated AMPK and CAR-mediated trancactivation of the phenobarbital-responsive enhancer module in HepG2 cells. Conversely, the reporter activity induced by the ectopic CAR was almost completely suppressed by co-transfection with the miR-122 mimic RNA. GFP-tagged CAR was expressed in the cytoplasm in addition to the nucleus in the majority of HuH-7 cells in which miR-122 was highly expressed. Co-transfection of the mimic or the inhibitor RNA for miR-122 further increased or decreased, respectively, the number of cells that expressed GFP-CAR in the cytoplasm. Taken together, these results suggest that phenobarbital-mediated down-regulation of miR-122 is an early and important event in the AMPK-dependent CAR activation and transactivation of its target genes.

## Introduction

Phenobarbital (PB) is commonly used as a sedative and antiepileptic drug. It elicits a multitude of effects in the liver, including the induction of drug-metabolizing enzymes as well as transporters, and it also exerts a non-genotoxic tumor promoter activity in experimental animals [1]. It has been demonstrated that a nuclear receptor, constitutive andorostane receptor (CAR), plays a pivotal role in these effects [2], [3]. The function of this transcription factor is regulated by its distribution between the cytosolic and the nuclear compartments. Namely, CAR is retained in the cytoplasm in the stationary phase and translocates into the nucleus upon a stimulus such as PB. Once there, it binds the 51 bp *cis*-element, the so-called PB-responsive enhancer module (PBREM) which is present in the 5′-upstream region of target genes together with its heterodimer partner, the retinoid X-receptor, and thus transactivates the transcription of these genes [4]. Since PB is not a direct ligand of CAR, phosphorylation and/or dephosphorylation [5], [6] as well as interaction with a chaperone protein [7] have been suggested to be involved in the mechanism underlying CAR nuclear translocation. However, the overall machinery by which this nuclear receptor is activated is not clearly understood.

Cytochrome P450 (CYP) 2B family is comprised of genes which are mostly inducible by CAR-activators. The contribution of CYP2B6 to human drug metabolism has been thought to be quite limited; however, recent studies using selective substrate probes and specific antibodies indicate that this isoform is involved in the biotransformation of 8-25% of pharmaceuticals [8]. The inter-individual difference in CYP2B6 expression varies significantly [8]. This is due, at least partly, to the inducibility of CYP2B6. In addition, the gene expression of CYP3A4, the major hepatic CYP in humans that is involved in the metabolism of almost one half of the pharmaceuticals on the market, is also controlled by CAR [9]. Consequently, elucidation of the machinery underlying CAR activation and the transactivation of *CYP* genes is important to understand not only drug-drug interactions but also individual differences in adverse drug reactions.

It has been demonstrated that AMP-activated protein kinase (AMPK) is involved in PB-mediated CAR activation [10], [11]. AMPK is activated by an increase in the AMP/ATP ratio in the cell and acts as an energy sensor that is involved in the regulation of gluconeogenesis and lipid metabolism [12]. The phosphorylation of AMPK at Thr172 by the upstream kinase LKB1 is necessary for its activation and PB reportedly activates AMPK in an LKB1-dependent manner [13]. These observations indicate that xenobiotics utilize the machinery of physiological function that is naturally present in hepatocytes and thus induce drug metabolizing enzymes. However, the precise mechanisms by which PB activates AMPK and in tern AMPK activates CAR are still undetermined.

The microRNAs (miRNAs) comprise a class of small (21–23 nucleotides) non-coding RNA. Accumulating evidence indicates that miRNAs play a pivotal role in fundamental processes such as cell growth, phenotype and death, mainly through gene suppression that takes place in a post-transcriptional manner [14]. In animal cells, most miRNAs bind a complementary sequence that resides in the 3′-untranslated region (3′-UTR) and thus induce the degradation or inhibit the translation of the target mRNA. It is postulated that each miRNA regulates up to 100 different mRNAs and that more than 10,000 mRNAs appear to be directly regulated by miRNAs [14]. However, the current knowledge of xenobiotic-mediated changes in miRNA levels, as well as miRNA function in drug metabolism is unfortunately quite limited [15]. MiR-122 is expressed in a liver-specific manner and is a major hepatic miRNA, accounting for more than 70% of total miRNA expressed in the liver [16]. Although it has been shown that miR-122 plays an important physiological role in lipid metabolism and liver development [16], it is likely there are also as yet unknown functions carried out by this miRNA in view of both its amount and the specificity of its liver expression pattern.

Our preliminary miRNA microarray experiments which preceded the present study suggested that PB induces a down-regulation of miR-122 in the mouse liver. Moreover, it has been reported [17] that miR-122 inhibition by systemic administration of an antisense oligonucleotide against miR-122 promotes the activation of hepatic AMPK. These observations prompted us to hypothesize that PB down-regulates miR-122, which in turn activates AMPK, leading to CAR activation and subsequently to the induction of *cyp2b* transactivation. The present study, by verifying this hypothesis, demonstrates that xenobiotics induce a change in the specific miRNA level, an effect which is involved in the induction of drug-metabolizing enzymes.

## Results

### PB induces a decrease in miR-122

**Figure 1:**
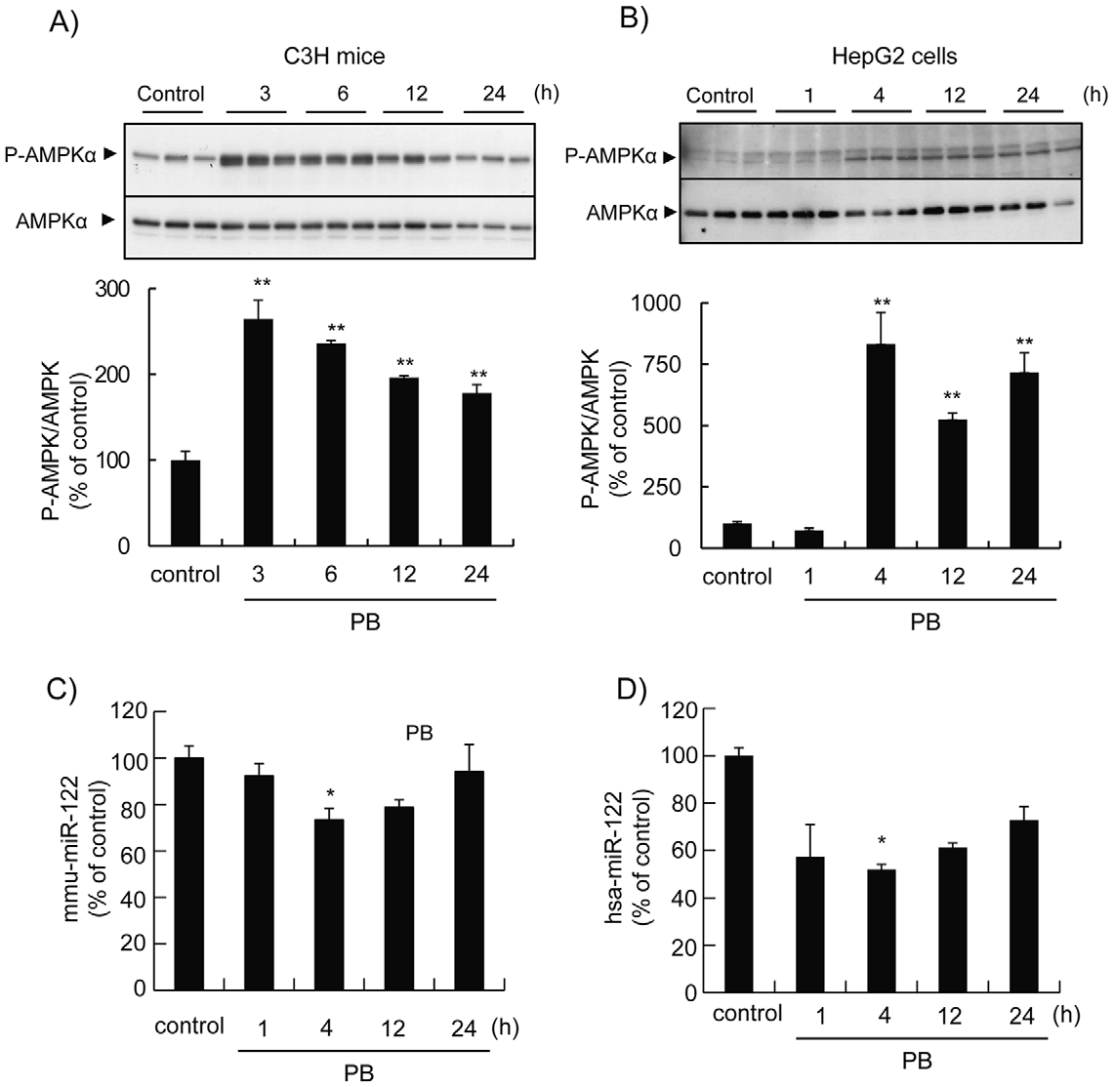
PB induces reciprocal changes in AMPK activation and miR-122 down-regulation. *A* and ***C***, Male C3H/HeN mice were treated with PB (100 mg/kg, *ip*.) and sacrificed times as indicated. ***B*** and ***D***, HepG2 cells were treated with 1 mM PB for times as indicated. Activated AMPK protein levels in the post mitochondrial fraction of the liver (***A***) and total cell lysate (***B***) were determined by Western blot analysis using phospho-AMPKα (Thr 172) specific antibody. The blot was sequentially probed with the AMPKα antibody. The closed arrow heads indicate phospho-AMPKα (P-AMPKα)- and total AMPKα-derived bands. Densitometric data from phospho-AMPKα were normalized by total AMPKα and a relative increase is shown. Total RNA samples from mouse liver (***C***) and HepG2 cells (***D***) were subjected to quantitative RT-PCR using a TaqMan probe for mouse (mmu-) and human (hsa-) miR-122, respectively. Data were normalized with the respective U6 snRNA. Data represented are the % of the control group (n = 3). *, *p*<0.05.

**Figure 2:**
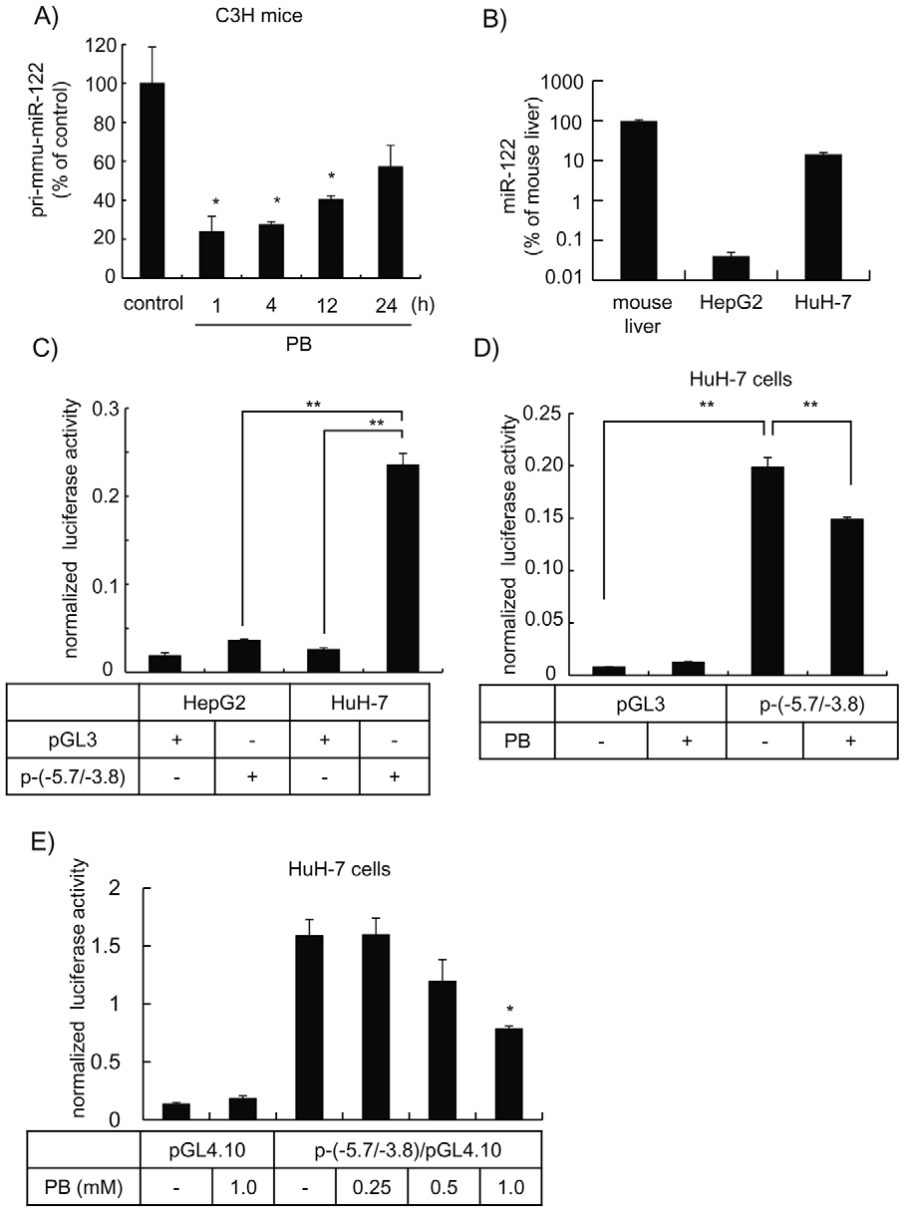
PB induces the down-regulation of miR-122 at the transcriptional level. ***A***, Total RNA from liver of mice treated with PB (100 mg/kg, *ip.*) was subjected to quantitative RT-PCR using a TaqMan probe for pri-miR-122. Data normalized with the respective U6 snRNA are presented as the % of control group (n = 3). *, *p*<0.05. ***B***, Total RNA samples from mouse liver, HepG2 and HuH-7 cells were subjected to quantitative RT-PCR using a TaqMan probe for mouse or human miR-122. Data were normalized with respective U6 snRNA and are presented as the % of mouse liver (n = 3). ***C***, HepG2 and HuH-7 cells seeded in 24-well multi-dish (1.5×10^5^ cells/well) were transfected with 0.9 μg of either pGL3 basic or p-(−5.7/−3.8) vector along with 0.1 μg phRL-TK vector. Firefly luciferase activity determined 48 h after the transfection was normalized with respective *Ranilla* luciferase activity (n = 3). **, *p*<0.01. ***D***, HuH-7 cells seeded in 24-well multi-dish were transfected either with 0.9 μg of either pGL3 basic or p-(−5.7/−3.8) vector along with 0.1 μg phRL-TK vector and were treated with 1 mM PB 12 h later. Firefly luciferase activity determined 48 h after the transfection was normalized with respective *Ranilla* luciferase activity (n = 3). **, *p*<0.01. ***E***, HuH-7 cells seeded in 96-well multi-dish (3×10^4^ cells/well) were transfected with 0.1 μg of either pGL4.10 or p-(−5.7/−3.8)/pGL4.10 vector along with 0.01 μg pGL4.74 and 0.09 μg pcDNA3.1 vectors and were treated with PB at concentrations illustrated 12 h later. Firefly luciferase activity which was determined 48 h after the transfection was normalized with respective *Ranilla* luciferase activity (n = 4). *, *p*<0.05.

It was demonstrated that PB induces sustained activation of AMPK in the murine liver [11] as well as human hepatocytes [10]. Fig. 1-A and C demonstrate changes in the miR-122 levels in mouse liver during the course of the AMPK activation induced by PB. The treatment of mice with PB at a dose (100 mg/kg, *ip*.), which is sufficient to induce the expression of the *cyp2b10* gene [11], significantly induced the activation of AMPK in the mouse liver, as determined by Thr172 phosphorylation of AMPKα. The AMPK activation by PB peaked within 3 h of the treatment and gradually decreased thereafter (Fig. 1-A). The mature miRNA levels of mouse origin (mmu-miR-122) were determined by real-time RT-PCR using the miR-122 specific reverse transcription primer and TaqMan probe. The treatment of mice with PB significantly decreased the hepatic mmu-miR-122 levels (Fig. 1-C). PB decreased the major hepatic miRNA to approximately 70% of the basal level 4 h after injection. The mmu-miR-122 level slowly returned to the basal level over 24 h. PB induced the significant and sustained activation of AMPK in human hepatoma HepG2 cells as well (Fig. 1-B). The maximum activation was observed 4 h after PB. Under these experimental conditions, PB induced a decrease in hsa-miR-122 in HepG2 cells, which contain very low amounts of this miRNA compared to hepatocytes (Fig. 2-B and [18]), as early as 1 h after treatment. The most significant decrease in hsa-miR-122 was observed 4 h after PB in these cells. These results suggest that PB induced the activation of AMPK and down-regulation of miR-122 in an inversely correlated manner in the mouse liver as well as HepG2 cells.

The miRNA level is mainly controlled by its transcription of the encoded gene or mature processes. The *mir-122* gene is independently transcribed, driven by its own promoter [18], [19]. Because mature miRNA is relatively stable, it was expected that the initial transcript of *mir-122* gene should be decreased more extensively if down-regulation of miR-122 by PB is caused at the transcriptional step. Therefore, the effect of PB on primary miR-122 (pri-miR-122) was determined by real-time RT-PCR specific for pri-miR-122. PB treatment of the mouse induced a considerable decrease in hepatic pri-miR-122. The pri-mir-122 level was decreased to approximately 25% of the basal one as early as 1 h (Fig. 2-A) after PB. A significant decrease in the pri-miR-122 level was observed between 1 to 12 h after the treatment and tended to return to the basal level gradually.

Down-regulation of miR-122 is frequently observed in hepatocellular carcinoma tissues [19]. The *MIR-122* promoter residing between −5.3 to −4.8 kb upstream of miR-122 precursor was characterized and shown to be involved in the down-regulation during the carcinogenesis [19] as well as hepatocyte differentiation [18]. The p-(−5.7/−3.8) reporter vector containing the promoter region 5′-upstream of the human *MIR-122* gene was used to assess the transcriptional activity of miR-122 [19]. In HepG2 cells in which the expression of miR-122 is approximately 0.04% of the mouse liver (Fig. 2-B), transfection of the miR-122 reporter vector resulted in only a trace increase in transcriptional activity (Fig. 2-C). On the other hand, a considerable increase in the promoter activity was observed in HuH-7 cells (Fig. 2-C), which express higher levels of miR-122, estimated at 14% of the mouse liver (Fig. 2-B). Treatment of HuH-7 cells with PB promoted a significant decrease in the reporter activity of the *MIR-122* promoter (Fig. 2-D). The suppressive effect of PB on the *MIR-122* promoter appeared in a concentration-dependent manner and 1 mM PB decreased approximately 50% of the activity in the reporter assay using the p-(−5.7/−3.8)/pGL4.10 vector which exhibits reduced non-specific expressions (Fig. 2-E). These results suggest that PB induces a decrease in miR-122 at the transcriptional level.

### Down-regulation of miR-122 induces AMPK activation

**Figure 3:**
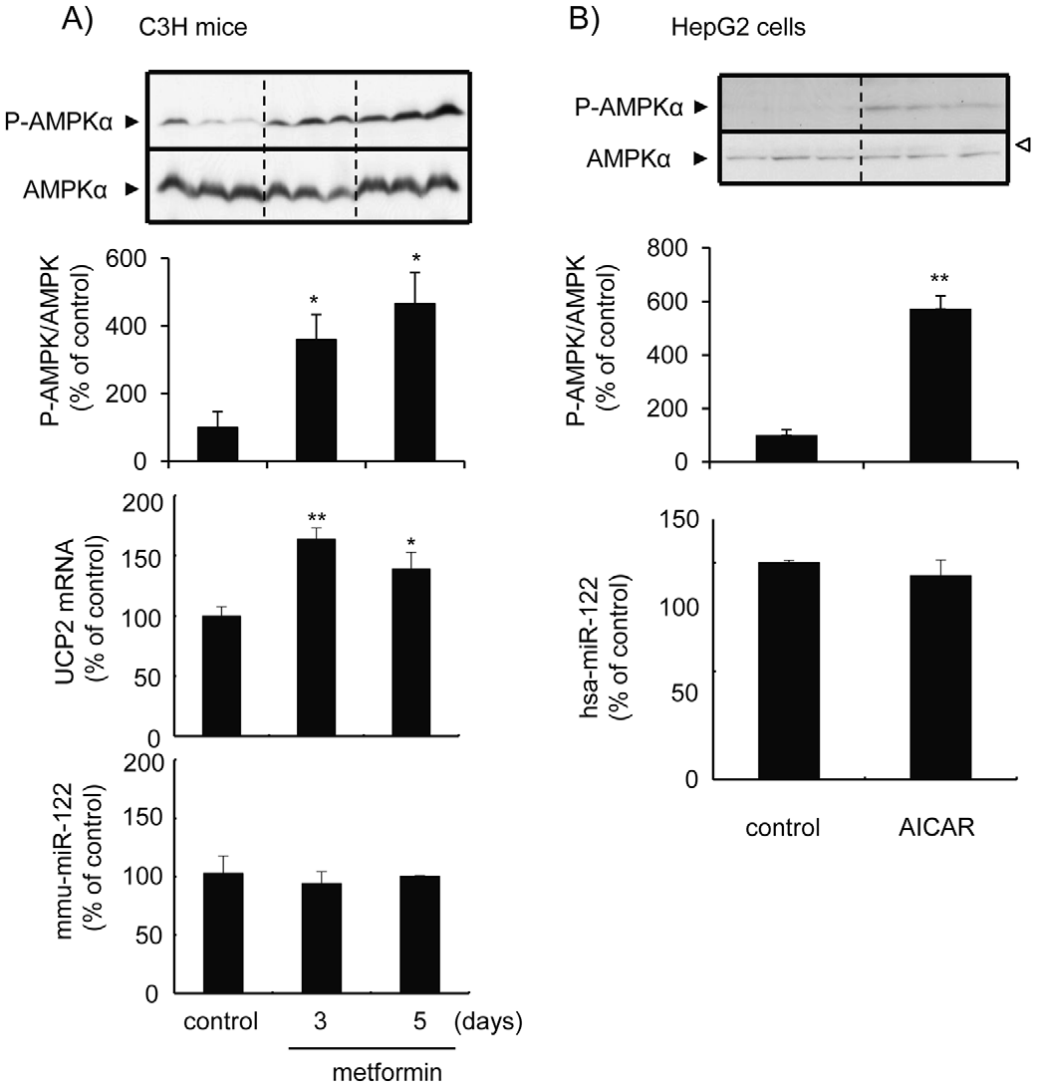
AMPK activation does not cause a miR-122 down-regulation. *A* , Mice were treated with metformin (250 mg/kg, *ip.*) twice a day at a 12 h-interval for 3 days or 5 days and were sacrificed 12 h after the final treatment. Activated and total AMPK levels in the liver of mice were determined by Western blot analysis using anti-phospho-AMPKα (Thr 172) and AMPKα specific antibodies, respectively (the top panel). Levels of phospho-AMPKα was normalized with respective AMPKα and are illustrated as the % of control group (the second panel). UCP2 mRNA (the third panel) and miR-122 (the last panel) levels were determined by the quantitative RT-PCR, were normalized with β-actin and the U6 snRNA, respectively, and are illustrated as the % of control group. *, *p*<0.05; **, *p*<0.01. ***B***, HepG2 cells were treated with AICAR (1 mM) for 24 h. Activated and total AMPK levels were measured by Western blot analysis using phospho-AMPKα (Thr 172) and AMPKα specific antibodies, respectively (the top panel). The open arrow head illustrated on the right indicates a nonspecific band. Levels of phospho-AMPKα was normalized with respective AMPKα and are illustrated as the % of control group (the middle panel). MiR-122 levels were determined by the quantitative RT-PCR, were normalized with the respective U6 snRNA and are illustrated as the % of control group (the last panel).

Metformin is a biguanide anti-hyperglycemic agent which activates AMPK, through its upstream kinase molecule LKB1 [20] and/or inhibition of complex I of the mitochondrial respiratory chain [21]. Treatment of mice with metformin twice a day for 3 or 5 days significantly increased AMPK phosphorylation as well as the transcripts of UCP2 (Fig. 3-A), a gene which is induced by AMPK-mediated signaling, indicating that the treatment did in fact activate the AMPK and its down-stream molecules in the liver. However, this protocol of metformin treatment apparently had no effect on the miR-122 levels in the mouse liver (Fig. 3-A). Treatment of HepG2 cells with 5-aminoimidazole-4-carboxamide 1-β-D-ribonucleoside (AICAR), another typical AMPK activator, which action is caused by mimicking an increased AMP/ATP ratio, induced the AMPK phosphorylation but apparently showed no effect on the miR-122 level (Fig. 3-B).

**Figure 4:**
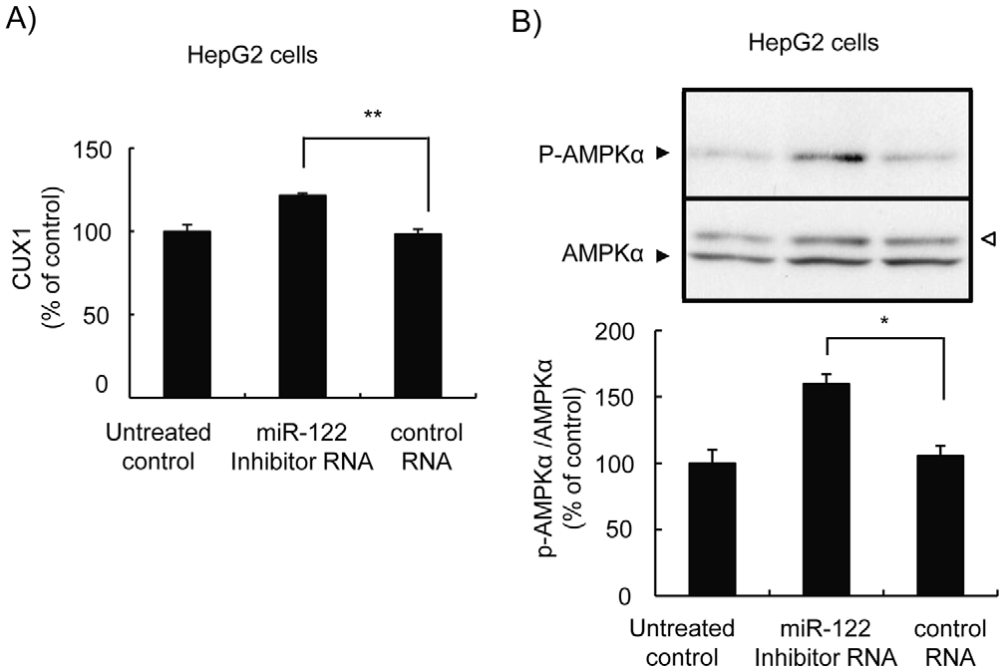
Inhibition of miR-122 induces AMPK activation in HepG2 cells. Cells were transfected either with the miR-122 inhibitor or the control RNA (20 pmol). ***A***, CUX1 mRNA levels 24 h after transfection were determined by quantitative RT-PCR. Data were normalized with respective β-actin and are illustrated as the % of untreated cells (n = 3). **, *p*<0.01. ***B***, Activated and total AMPK levels 24 h after transfection were determined by Western blot analysis using anti-phospho-AMPKα (Thr 172) and AMPKα specific antibodies, respectively (upper panel). The open arrow head illustrated on the right indicates a nonspecific band. The values obtained from band intensity measurements of the phospho-AMPKα were normalized with those of AMPKα and are illustrated as the % of untreated control (n = 3). *, *p*<0.05.

Experiments were then conducted to determine whether an inhibition of miR-122 induces AMPK activation using miR-122 inhibitor RNA, a 2′-methoxy modified and single stranded RNA complementary to hsa-miR-122 which was designed to repress function of the target miRNA. Transfection of HepG2 cells with the control RNA showed virtually no change in the gene expression of cut-like homeobox 1 (CUX1), a transcriptional repressor in which the mRNA is targeted by miR-122 [18]. Transfection of the miR-122 inhibitor RNA resulted in a significant increase in CUX1 gene expression (Fig. 4-A), indicating that the function of miR-122 is in fact repressed. Under these experimental conditions, the miR-122 inhibitor RNA significantly induced AMPK phosphorylation (Fig. 4-B). These results strongly suggest that the down-regulation of miR-122 induces AMPK activation.

### The miR-122 level affects CAR-mediated PBREM transactivation

**Figure 5:**
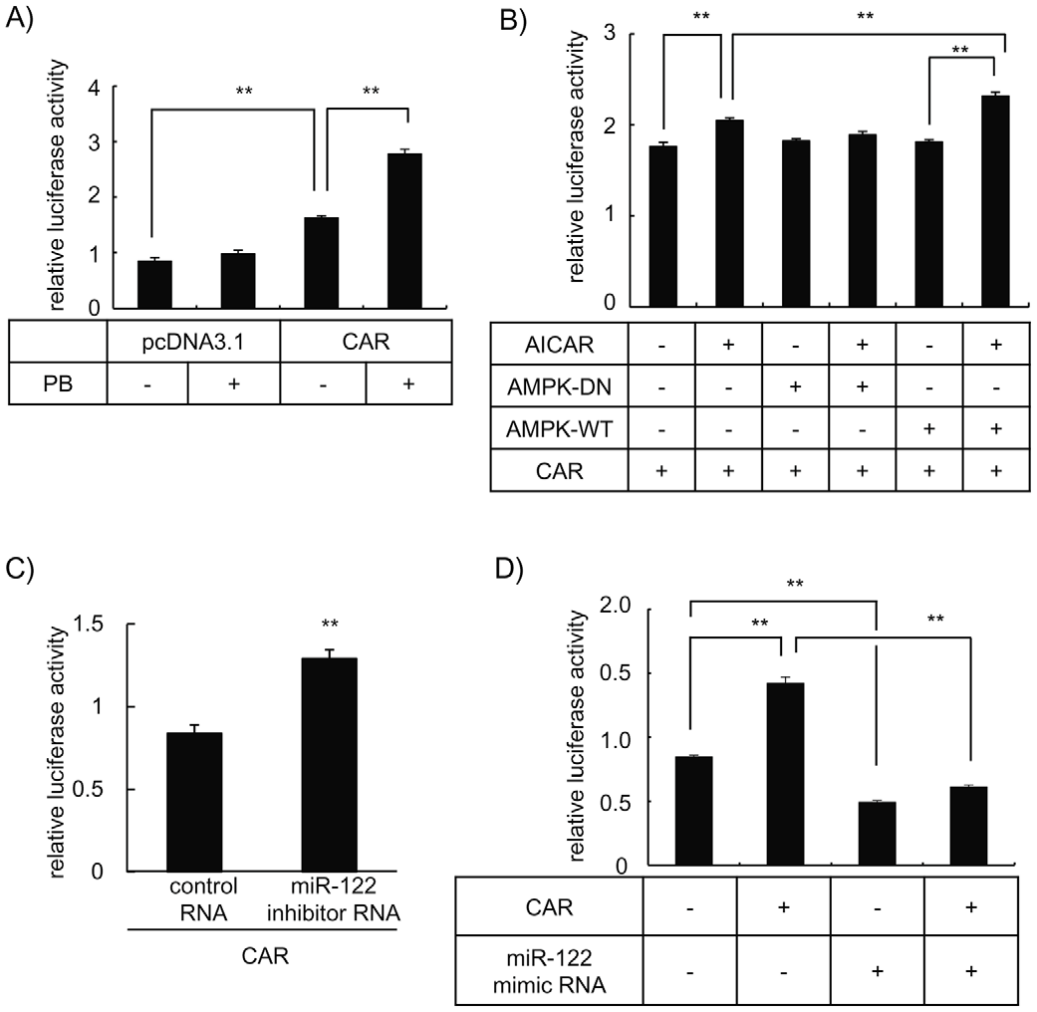
The inhibitor or mimic RNA for miR-122 induces activation or suppression, respectively, of CAR-mediated PBREM transactivation in HepG2 cells. *A* , Cells seeded in 24-well multi-dish were transfected either with CAR/pcDNA3.1 or empty pcDNA3.1 (0.2 μg) in conjunction with the PBREM/pGL3-120 (0.7 μg) and pRL-TK vector (0.1 μg). Cells were treated with PB (1 mM) 12 h after transfection. The luciferase activity was determined 48 h after PB treatment. Data were normalized with the respective *Renilla* luciferase activity (n = 3). *, *p*<0.01. ***B***, Cells were transfected either with AMPKα2ΔC T172A/pcDNA3.1 (AMPK-DN), AMPKα2/pcDNA3.1 (AMPK-WT) or empty pcDNA3.1 (0.7 μg) in conjunction with the PBREM/pGL3-120 (0.2 μg), phRL-TK (0.05 μg) and CAR/pcDNA3.1 (0.05 μg) vectors. Cells were treated with AICAR (1 mM) 12 h after transfection. The luciferase activity was determined 48 hr after AICAR treatment. Data were normalized with the respective *Renilla* luciferase activity (n = 3). *, *p*<0.01. ***C***, Cells were transfected either with the miR-122 inhibitor or the control RNA (40 pmol) in conjunction with the PBREM/pGL3-120 (0.12 μg), pRL-TK (0.04 μg) and CAR/pcDNA3.1 (0.04 μg) vectors. The luciferase activity was determined 48 hr after transfection. Data were normalized with the respective *Renilla* luciferase activity. (n = 3). *, *p*<0.05. ***D***, Cells were transfected with the miR-122 mimic RNA or the control RNA (10 pmol) in conjunction with CAR/pcDNA3.1 or pcDNA3.1 vector (0.04 μg) as well as the PBREM/pGL3-120 (0.12 μg) and pRL-TK vector (0.04 μg). The luciferase activity was determined 48 h after transfection. Data were normalized with the respective *Renilla* luciferase activity (n = 3). **, *p*<0.01.

In HepG2 cells, endogenous CAR gene expression is restricted and the ectopic expression results in the accumulation of this transcription factor in the nuclear compartment. Therefore, such an *in vitro* system is not appropriate to observe the translocation of CAR from the cytoplasm to the nuclear compartment, which is an event necessary for CAR function, *i.e.*, the PBREM-mediated gene transactivation. On the other hand, as shown in Fig. 5-A, forced expression of CAR in HepG2 cells increased the reporter activity of PBREM and treatment with PB further stimulated the ectopically expressed CAR function. In this *in vitro* system, AICAR stimulated the PBREM transcriptional activity, which was canceled or augmented in the presence of the dominant negative mutant or wild type of the AMPKα expression plasmid, respectively (Fig. 5-B). These results suggest that AICAR activates CAR function in an AMPK-dependent manner, which are consistent with our previous study [11] using *in vivo* system in which the PBREM-reporter plasmid was transfected into mouse liver. Collectively, these results indicate that PB-stimulated CAR function can be evaluated, at least in part, by using the CAR-expressing HepG2 cells.

The effects of loss or gain of function of miR-122 on PBREM transcriptional activity were evaluated using this ectopic CAR-expressed *in vitro* system. Transfection of the miR-122 inhibitor RNA significantly augmented the CAR-stimulated PBREM-reporter activity (Fig. 5-C), suggesting that the suppression of miR-122 function activates AMPK and in turn induces the CAR-mediated PBREM transactivation. In contrast, the PBREM reporter activity induced by the ectopic expression of CAR was almost completely suppressed by co-transfection of the miR-122 mimic RNA (Fig. 5-D). Therefore, these results suggest that miR-122 level affects CAR-mediated PBREM transactivation. It is interesting to note that a significant decrease in the basal level of PBREM reporter activity was observed in cells transfected with the miR-122 mimic RNA in the absence of the ectopic CAR expression. It is possible that miR-122 may promote a decrease in PBREM reporter activity independent of CAR in addition to the CAR-mediated mechanism.

### MiR-122 regulates the CAR distribution pattern

**Figure 6:**
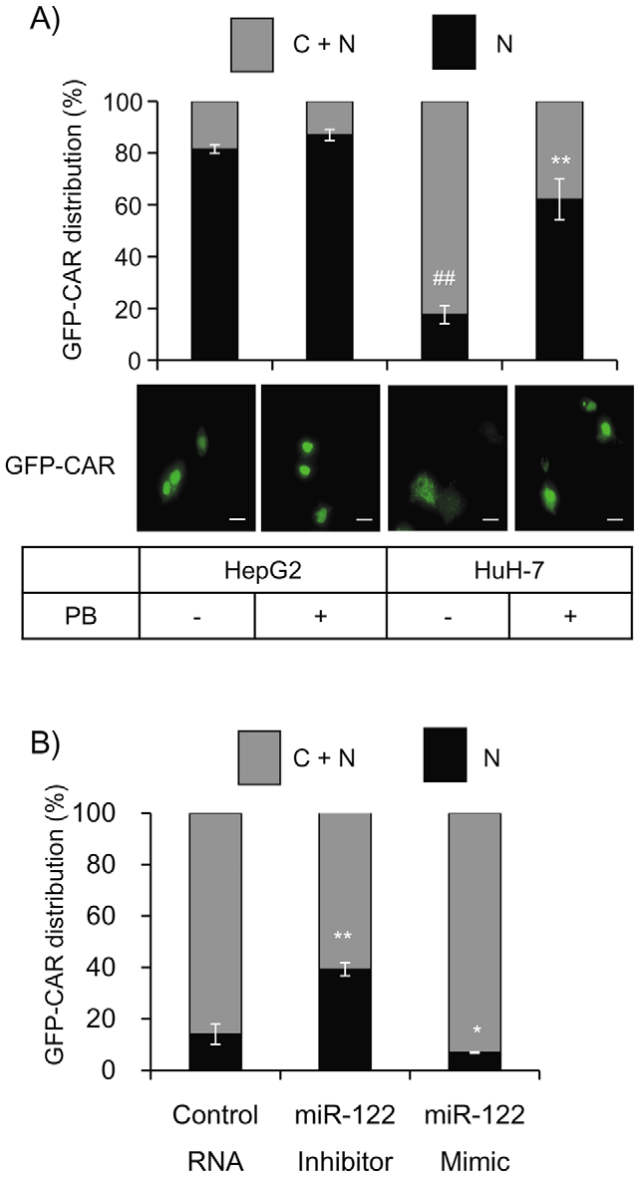
Modulation of miR-122 induces changes in GFP-CAR distribution in HuH-7 cells. *A* , HepG2 and HuH-7 cells were transfected with CAR/pQBI25. Cells were treated with PBS or PB 24 h after transfection and fixed 4 h after the treatment with 2% paraformaldehyde. Subcellular localization of the GFP fusion protein was observed by Keyence BZ-8000 microscope using a x20 objective lens (lower panel; scale bar, 20 μm). The distribution pattern were scored for at least 100 transfected cells, which were randomly observed, and classified into 2 categories; C+N, equal distribution to the nuclear and the cytoplasmic compartments; N, the nuclear dominant distribution. Data are illustrated as the % of total cells (n = 3). ##, *p*<0.01 vs the vehicle treated HepG2 cells; **, *p*<0.01 vs the vehicle treated HuH-7 cells. ***B***, HuH-7 cells were transfected either with the miR-122 inhibitor, the mimic or the control RNA (20 pmol) in conjunction with CAR/pQBI25 vector (0.2 μg). Cells were fixed 48 h after transfection and the subcellular localization of the GFP-derived fluorescence was monitored as described above. Data are illustrated as the % of total cells (n = 3). *, *p*<0.05; **, *p*<0.01 vs cells transfected with the control RNA.

As described above, unlike most other hepatoma cell lines including HepG2 cells, HuH-7 cells express a higher level of miR-122 [18], [22], *i.e.* approximately 14% of that expressed in mouse liver (Fig. 2-B). Therefore, we transfected GFP-tagged CAR into HuH-7 cells to observe its distribution pattern in cells. In HepG2 cells, expression of ectopic GPP-CAR resided mostly in the nucleus and PB has apparently no effect on its distribution. In contrast, 82% of HuH-7 cells expressed GFP-CAR in the cytoplasm in addition to the nucleus and the PB treatment significantly increased the number of cells displaying fluorescent signals in the nucleus (62%, Fig. 6-A). Furthermore, co-transfection of the miR-122 inhibitor RNA with the GFP-CAR plasmid increased and the mimic RNA inversely decreased the number of cells displaying nuclear signals (Fig. 6-B). These results indicate that GFP-CAR was preferentially distributed in the cytoplasm of miR-122 enriched HuH-7 cells and suggest that there exists a miR-122-dependent machinery which is involved in the cytosolic retention of CAR.

## Discussion

Because a single miRNA molecule can regulate plural targets and each target in turn can be regulated by multiple miRNAs simultaneously, these small RNAs are multifunctional and are implicated in diverse cellular events [14]. It is possible therefore that miRNA is involved in drug-mediated changes in gene expression, although only limited information on this sort of effect is available at the present time [15]. The current study demonstrates that PB induces down-regulation at the transcriptional level of liver enriched miR-122, which is implicated in hepatic differentiation and function. The changes in miRNA expression induced by drugs may provide important means for understanding its pharmacological effects, adverse drug reactions and effect on the drug-metabolizing enzymes.

The present study sought to demonstrate a link between miR-122 and AMPK based on the fact that PB induced reciprocal changes in the miR-122 levels and AMPK activity in both mouse liver and HepG2 cells. It is important to note that a 30% decrease in mature miR-122 in the PB-treated mouse liver may elicit significant biochemical effects because it accounts for more than 70% of the total hepatic miRNA [16]. Transfection of the inhibitor RNA, which specifically blocks the miR-122 function, induced AMPK activation in HepG2 cells. These results are consistent with a report [17] showing that *in vivo* treatment with an antisense oligonucleotide for miR-122 induced hepatic AMPK activation. On the other hand, AMPK activation induced by metformin or AICAR had little effect on miR-122 levels, suggesting that miR-122 plays a role in the suppression of AMPK activity. The miR-122 mimic RNA effectively suppressed the CAR-mediated PBREM transactivation in HepG2 cells and increased the cytoplasmic distribution of GFP-CAR in HuH-7 cells. The miR-122 inhibitor RNA conversely induced the PBREM transactivation and increased the nuclear distribution of GFP-CAR. Consequently, all of these data suggest that PB releases AMPK from suppressive control through the down-regulation of miR-122, resulting in the nuclear translocation of CAR and *CYP2B* transactivation. It is reported [5] that dephosphorylation of Thr 38 of human CAR, a target site of protein kinase C (PKC), induces the nuclear translocation. There is no information that connects miR-122 and PKC at the present time; however, elucidation of relationship between changes in the miRNA level and phosphorylation states of CAR would be attractive and hopefully shown in the following project.

The precise mechanism that governs the inhibitory effect of miR-122 on AMPK activation has not been elucidated in this study. AMPK is activated by the change in the AMP/ATP ratio as well as upstream kinases such as LKB1. It has been reported [13] that LKB1 is involved in PB-induced AMPK activation, suggesting that miR-122 may regulate LKB1 function. LKB1 is allosterically activated by the formation of a ternary complex with the pseudokinase STRAD and the scaffolding protein MO25 [23]. It was reported [24] that miR-451 negatively regulates LKB1 activity by binding directly to a 3′-untranslated region of MO25 mRNA and suppressing its expression. These observations imply a miR-122-mediated mechanism regulating LKB1 activity. However, the DNA array analysis we performed using mRNA from PB-treated mouse liver failed to reveal any candidate for involvement in the AMPK pathway (data not shown). It is possible that PB-mediated miR-122 down-regulation is not directly linked to AMPK or its upstream signaling molecules. Further progress in understanding miRNA function will be required to ultimately reveal the mechanistic relationship between miR-122 and AMPK.

*Mir-122* is an intergenic miRNA which expression is controlled by its own promoter. The promoter region is reportedly regulated by the liver-enriched transcription factors (LETFs), such as CCAAT/enhancer-binding protein α (C/EBPα) [18], [19], hepatocyte nuclear factor (HNF) 1α [18], HNF3 [18], [22] and HNF4α [18], which are tightly correlated with hepatocyte differentiation and function. A number of reports have indicated that these LETFs are involved in the gene expression of drug metabolizing enzymes [25]. In addition, there is evidence [26], [27] which indicates that PB induces the activation of HNF4α, suggesting that a simple scenario in which PB collectively inhibits the LETFs is unlikely. It has been reported that the function of C/EBPα including the transactivation of *mir-122* gene is activated by GSK3β [19], [28], which is phosphorylated and inactivated by extracellular signal-regulated kinases (ERK1/2) [29]. A high concentration of PB is reportedly capable of activating ERK1/2 *in vitro* [30], suggesting that deterioration of C/EBPα transactivity could be involved in miR-122 down-regulation by PB. However, an inhibitor of ERK1/2 pathway (U0126) had apparently no effect on the PB-mediated down-regulation of the transactivation of the *mir-122* promoter in HuH-7 cells (data not shown). Moreover, it has been demonstrated that activated ERK1/2 can sequester CAR in the cytoplasm [6]. Further study is needed to clarify the PB-induced intracellular signaling that is connected to the down-regulation of the constitutive miR-122 transcription.

In conclusion, the present study demonstrated that xenobiotics can modulate the miRNA level which in turn activates CAR in an AMPK-dependent manner. The results presented in this study also suggest that the PB-mediated down-regulation of miR-122 is an early and key event linked to the CAR-mediated transactivation of target genes. Xenobiotic-mediated changes in the miRNA level are not well understood yet, but have come to be increasingly demonstrated [31]–[35]. The present study extends these earlier studies and shows that the xenobiotic-mediated changes in miRNA are tightly connected to drug-drug interactions. In addition to the function as a xenosenser, CAR also has an endobiotic role that impacts energy homeostasis through the regulation of glucose and lipids metabolism [36]. Therefore, the results obtained in the current study suggest a new role for the liver-specific miRNA in drug-induced tissue toxicity.

## Materials and Methods

### Materials

Anti phospho-AMPKα (Thr-172) and AMPKα antibodies were purchased from Cell Signaling Technology, Inc. (Beverly, MA). The pGL3-basic, pGL4.10, pRL-TK, phRL-TK and pGL4.74 vectors were obtained from Promega (Madison, WI). The pcDNA3.1 vector and all probes for real-time PCR were obtained from Life Technologies (Rockville, MD). Anti-hsa-miR-122 miScript miRNA inhibitor (MIN0000421), syn-hsa-miR-122 miScript miRNA mimic (MSY0000421) and the AllStars Negative Control siRNA were purchased from Qiagen (Valencia, CA). The miR-122 inhibitor (inhibitor RNA) is a chemically synthesized and modified single-strand RNA (5′ UGGAGUGUGACAAUGGUGUUUG) which specifically inhibits endogenous miR-122 function after transfection into cells. The miR-122 mimic (mimic RNA) is a synthetic double strand RNA (5′ UGGAGUGUGACAAUGGUGUUUG) which mimics mature endogenous miRNA after transfection. AllStars Negative Control siRNA (control RNA) is a scramble oligo which has no homology to any known mammalian gene. It has been validated using Affymetrix GeneChip arrays and a variety of cell-based assays and shown to ensure minimal nonspecific effects on gene expression and phenotype [37].

### Animals and cell cultures

All animal experiments were conducted under NIH guidelines for the care and use of laboratory animals and were approved by the Showa University Institutional Animal Care and Use Committee (Permit Number: 21035). Male C3H/HeN mice (7 weeks of age) were purchased from Sankyo Labo Service (Tokyo, Japan). Animals were intraperitoneally administered PB (100 mg/kg). HepG2 and HuH-7 human hepatoma cells obtained from the Riken Cell Bank (Tsukuba, Japan) were cultivated in DMEM and RPMI1640, respectively, supplemented with 10% fetal calf serum, 20 mM HEPES and antibiotics in a humidified 5% CO_2_ atmosphere at 37°C.

### Plasmids

The expression plasmids for rat CAR (CAR/pcDNA3.1), GFP tagged rat CAR (CAR/pQBI25), rat AMPKα2 (AMPKα2/pcDNA3.1) and a dominant negative mutant of rat AMPKα2 (AMPKα2ΔC T172A/pcDNA3.1) have been described previously [11]. A luciferase reporter construct p-(−5.7/−3.8) containing the −5645 to −3726 bp region of the human *mir-122* gene (chr18: 54263641–54265560) in the pGL3-basic vector [19] was kindly donated by Shi-Mei Zhuang (Sun Yat-Sen University, China). The p-(–5.7/–3.8) vector was digested by *Kpn*I and *Xho*I, resulting a 1919 bp fragment that was inserted into the pGL4.10 vector to obtain p-(–5.7/–3.8)/pGL4.10 vector. To construct the PBREM reporter vector, the proximal promoter region (–123 to +18 bp) of the *cyp2b10* gene was PCR-amplified and inserted into the *Bgl*II/*Hind*III site of a pGL3 vector (pGL3-120). A double-strand DNA fragment containing the 51 bp PBREM of the *cyp2b10* gene was obtained by PCR and inserted upstream of the proximal promoter of the pGL3-120 vector (PBREM/pGL3-120).

### Transfection and reporter assay

Lipofectamine 2000 (Life Technologies) was used for transfection of the plasmid alone or together with RNA oligonucleotides. When transfecting HepG2 cells seeded in a 24 well multi-dish with plasmid alone, 1.0 μg DNA and 2 μL Lipofectamine 2000 were used. For HuH-7 cells, 0.8 μg DNA and 1 μL Lipofectamine 2000 were employed unless otherwise specified. When transfecting DNA together with the RNA oligonucleotides, 0.2 μg DNA and 20 pmol RNA were combined with 1.5 μL Lipofectamine 2000. Cell lysate was subjected to a dual luciferase assay system (Promega). Firefly luciferase activity was normalized to the respective *Ranilla* luciferase activity.

### Western blot analysis

Liver specimens were homogenized with the lysis buffer (20 mM Tris-HCl, pH 7.6, containing 150 mM NaCl, 1% NP-40, 10 mM *p*-nitrophenylphosphate, 1 mM sodium orthovanadate, 10 mM sodium molybdate, 20 mM β-glycerophosphate and the protease inhibitor cocktail). The homogenate was centrifuged at 15,000 rpm for 20 min and the resulting supernatant was used for Western blot analysis. Cultured cells were washed and lysed by scraping them into boiling SDS-PAGE sample buffer. The lysates were further boiled for 5 min and briefly sonicated. Western blot analysis was carried out as described previously [11].

### Quantitative real-time reverse transcription-PCR

Total RNA isolated from frozen liver or cultured cells using an miRNeasy Mini Kit (Qiagen) was subjected to the reverse transcription (RT) reaction using High-Capacity cDNA Reverse Transcription Kits (Life Technologies) for the analysis of mRNA or primary miRNA (priRNA). The cDNA samples were used for the duplex TaqMan real-time PCR using a FAM labeled probe for the target mRNA and a VIC labeled β-actin probe. Assay ID of the probes used in this study were as follows: mouse UCP2, Mm00627597_m1; human CUX1, Hs00738851_m1; mouse actin, Mm00607939_s1; human actin:Hs99999903_m1. To analyze the mature form of miR-122, total RNA was reverse-transcribed with TaqMan MicroRNA Reverse Transcription Kits (Life Technologies) using a specific primer for miR-122. The resulting cDNA samples for priRNA and miRNA were subjected to a singleplex reaction of the real-time PCR using a probe for mouse pri-miR-122 (assay ID, Mm03306556_pri) or miR-122 (assay ID, 000445) of mouse and human origin. U6 small nuclear RNA (U6 snRNA, 001973) was determined in parallel for the reference. All data were analyzed by the ΔΔCt method.

### Statistical analysis

All data are provided as the mean ± S.E.M. The significance of the difference between the control and treated group(s) was assessed by Student's *t*-test or Dunnett's test for data from two or multiple groups, respectively. The one-way analysis of variance followed by Tukey's *post hoc* test was performed to compare data from multiple groups with each other. Values of p<0.05 were taken to be significant.
