# Correction: MicroRNA-122 Down-Regulation Is Involved in Phenobarbital-Mediated Activation of the Constitutive Androstane Receptor

**DOI:** 10.1371/annotation/75898a72-dfcb-4002-b749-8c04d78ba6e1

**Published:** 2013-01-14

**Authors:** Ryota Shizu, Sawako Shindo, Takemi Yoshida, Satoshi Numazawa

The authors would like to make readers aware that several of the figures in this article are duplicated from those included in the publication in Yakugaku Zasshi below:

Yakugaku Zasshi. http://www.ncbi.nlm.nih.gov/pubmed/223828352012;132(3):311-8.

Involvement of microRNA in the induction of drug-metabolizing enzymes.

Shizu R. http://www.ncbi.nlm.nih.gov/pubmed?term=Shizu%20R%5BAuthor%5D%26cauthor=true%26cauthor_uid=22382835, Numazawa S. http://www.ncbi.nlm.nih.gov/pubmed?term=Numazawa%20S%5BAuthor%5D%26cauthor=true%26cauthor_uid=22382835, Yoshida T. http://www.ncbi.nlm.nih.gov/pubmed?term=Yoshida%20T%5BAuthor%5D%26cauthor=true%26cauthor_uid=22382835. 

In particular, the HepG2 cell data in Figure 1, Figure 2A, Figure 4B and Figures 5A, 5C, 5D in the PLOS ONE article overlap with those reported in the publication in Yakugaku Zasshi. The remaining results and figures reported in the PLOS ONE article are original.

The authors apologize to the editors and readers for this overlap and for failing to adequately cite and acknowledge the Yakugaku Zasshi article in the submission to PLOS ONE. 

